# Effectiveness of Iterative Practice in Pediatric Simulation at a Community Hospital

**DOI:** 10.7759/cureus.105834

**Published:** 2026-03-25

**Authors:** Azadeh Fayazi, Siobhan Hopkins

**Affiliations:** 1 Pediatric Critical Care Medicine, Stanford University, Palo Alto, USA; 2 Pediatric Hospitalist Medicine, Stanford University, Palo Alto, USA

**Keywords:** community hospitals, pediatric cpr, resuscitation and simulation research in pediatrics, simulation in medical education, team dynamics

## Abstract

Objective

The goal of this study was to improve knowledge and teamwork within a limited simulation period. We hypothesized that implementing iterative practice will increase pediatric resuscitation knowledge and specific team behaviors by 10%.

Methodology

We implemented an in situ, multidisciplinary, high-fidelity simulation curriculum, in which the scenario was repeated. This study tracked improvements through three multiple-choice questionnaires. Data collected over two years were analyzed using McNemar’s test to compare paired binary variables and the Wilcoxon signed-rank test to compare paired ordinal variables.

Results

A total of 99 questionnaires were completed. Knowledge of indications for cardiopulmonary resuscitation in children improved by 12% (n = 12, p = 0.008); chest compression-to-ventilation ratio in adolescents increased by 16% (n = 16, p = 0.005); chest compression-to-ventilation ratio in children improved by 11% (n = 11, p = 0.03); and ventilation rate with an advanced airway increased after both simulations by 22% (n = 22, p < 0.001) and then by 13% (n = 13, p = 0.004). Statistically significant improvements were not observed for choosing the correct shockable rhythms. All team behaviors improved: information was “always” received by 81% (n = 80) of participants and increased to 94% (n = 93, p < 0.001); self-identification improved from 66% (n = 65) of participants to 87% (n = 86, p < 0.001); and leader identification was noted by 73% (n = 72) of non-leader participants and increased to 88% (n = 87, p < 0.001).

Conclusion

Iterative simulation practice effectively enhances proximal knowledge and pediatric resuscitation team performance in community hospitals. Future research should explore the simulation frequency needed to retain this knowledge and these skills.

## Introduction

The importance of high-fidelity simulation education for trainees has been well-established [1,2]. However, research on the use of simulation for continuing medical education in inpatient community settings and among practicing healthcare professionals remains limited [3]. Our study aims to address this gap by examining how simulation training in a community hospital setting, using iterative practice methods, can enhance pediatric providers’ knowledge and emergency preparedness. 

Community hospitals typically have smaller pediatric units with lower patient volume and acuity. Though children with severe critical illness are less likely to be admitted to such hospitals, acute deterioration and cardiac arrest can occur within their pediatric units, emergency departments (EDs), and operating rooms. More importantly, pediatric code teams in these hospitals often consist of interdisciplinary staff who lack pediatric-specific training or Pediatric Advanced Life Support (PALS) certification. 

Less experience in pediatric resuscitation risks patient outcomes. Studies have shown that lower-volume EDs have higher mortality among pediatric patients [4], and a low composite quality score evaluating adherence to PALS guidelines and teamwork [5]. Several studies have demonstrated the effectiveness of simulation in improving emergency preparedness for children in community settings [6,7]. Simulation training has also been shown to enhance decision-making and support long-term skill retention [8]. In addition, it increases knowledge and confidence among critical care providers [9] and can improve procedural performance, communication skills, medical knowledge, teamwork, and adherence to resuscitation protocols [8,10].

Iterative practice is a cyclical learning strategy in which a clinical scenario or procedure is repeated with small, targeted adjustments based on immediate facilitator feedback. In medical simulation, this repeated performance-feedback-correction cycle helps refine skills, correct errors, and build “muscle memory,” rather than relying on a single debriefing session at the end. This is similar to deliberate practice, which involves repetition until mastery is achieved. In the academic clinical setting, deliberate practice is more beneficial in achieving mastery of specific skills and goals, compared to traditional methods [11]. Given the limited time available with participants in a community setting, progressive improvement with iterative practice can be a more practical goal, especially with communication skills.

A multidisciplinary simulation curriculum was implemented for pediatric staff and physicians. Participants engaged in a pediatric scenario requiring cardiopulmonary resuscitation (CPR), followed by a facilitated debrief, a repeated scenario, and a final facilitated debrief. This study aimed to evaluate whether an iterative in-situ simulation model improves pediatric resuscitation knowledge and team behaviors among multidisciplinary providers in a community hospital. The hypothesis was that reviewing PALS guidelines during the scenario and debrief, combined with the opportunity to correct mistakes in a second simulation, would lead to a 10% improvement in PALS-specific knowledge and communication after each simulation session. 

## Materials and methods

Study design

The study was conducted at a community hospital with a 12-bed pediatric unit and an 8-bed Pediatric Intensive Care Unit (PICU) from January 2023 to October 2024. The hospital Institutional Review Committee approved the study. The in-situ simulations were run in the Pediatric Acute Care and Intensive Care Units. The participants were recruited during monthly pediatric resuscitation simulation education sessions. Most non-pediatric staff and physicians participated only in the first of the two simulation sessions, attending when a hospital-wide “Pediatric Code Blue Drill” was announced; most did not complete the questionnaires. 

**Figure 1:**
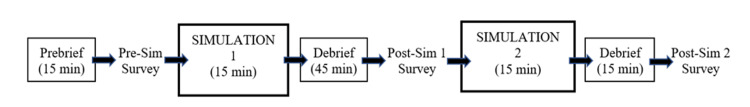
Iterative Practice Session Timeline Pre-sim: pre-simulation; Post-sim 1: post-simulation 1; Post-sim 2: post-simulation 2

Each learning session lasted two hours. After a 15-minute pre-brief, the first simulation (sim 1) was run over 12-15 minutes, followed by a 45-minute debrief based on the PEARLS framework (Figure 1) [12]. The facilitators were two pediatric physicians certified in simulation and debriefing. The debrief started by eliciting reactions, followed by a summarization of the clinical scenario by participants. Advocacy/inquiry, guided self-reflection, and directive feedback were techniques used to analyze participant actions and decisions. Clinical explanations for the relevant aspects of the PALS guidelines and the medical management in each case were reviewed to close knowledge gaps. After this first debrief, the exact same scenario was run again (sim 2), with some rotation of team roles. The session ended with a 10-15-minute debrief that mainly included participant reactions to the repetition, directive feedback, and take-away points. 

Scenarios were designed based on the objectives of the curriculum. Given the multidisciplinary nature of the simulation, the objectives were broad but mainly included the creation of an organized resuscitation team with a clear leader, and the fulfillment of code roles (e.g., chest compressor, recorder, cardiac coach, medication preparer), appropriate communication, and resuscitation of a pediatric patient per PALS guidelines. In 2023, the acute care scenario was bradycardia leading to cardiac arrest in an immunocompromised child (SimJunior®; Laerdal Medical, Wappingers Falls, NY, USA) with septic shock, and the PICU scenario was an adolescent (SimMan®; Laerdal Medical, Wappingers Falls, NY, USA) with respiratory failure secondary to pneumonia and pulmonary hypertensive crisis, progressing into cardiac arrest. In 2024, the scenario for both locations was ventricular tachycardia due to tricyclic antidepressant overdose in an adolescent (SimMan®). 

Our specific aims in this study were to increase pediatric CPR knowledge and skills and improve team dynamics. The knowledge questions had been iteratively pilot-tested in the previous year during similar multidisciplinary mock codes. This ensured adequate face and content validity, tailored to the specific educational objectives of the pediatric simulation curriculum. These questions pertained to the 2020 PALS guidelines and were the same in all three questionnaires (Table 1). The team dynamic questions were designed to be simple and straightforward for self-reporting and were only implemented in the post-sim 1 and post-sim 2 questionnaires (after learner participation in a simulation) (Table 1). The surveys were distributed using separate QR codes, which were scanned with participant smartphones.

**Table 1:** Survey Questions CC: chest compressions; Vent: ventilation; min: minute; PEA: pulseless electrical activity; SVT: supraventricular tachycardia

Domain	Questions
Knowledge	1. What is the ratio of compressions to ventilation in an adolescent in cardiac arrest with 2 rescuers? (Adolescent CPR)
	a. No ratio, continuous CC and ventilation
	b. 15:2
	c. 5:3
	d. 30:2
	2. What is the ratio of compressions to ventilation in an infant in cardiac arrest with 2 rescuers? (Child CPR)
	a. 5:3
	b. 30:2
	c. 15:2
	d. 15:1
	3. What is the ventilation rate in a child with an advanced airway during cardiac arrest? (Vent Rate w/ Advanced Airway)
	a. CC:2 vent ratio
	b. 10-12 breaths per min
	c. 12-15 breaths per min
	d. 20-30 breaths per min
	4. What is the indication for initiating CPR in a child? (CPR Indication)
	a. Heart rate <60
	b. Loss of consciousness
	c. Apnea
	d. Absence of pulse or bradycardia with poor perfusion
	5. Which of the following are shockable rhythms? (Shockable Rhythms)
	a. Ventricular fibrillation and pulseless ventricular tachycardia
	b. Ventricular tachycardia and asystole
	c. Ventricular fibrillation and PEA
	d. PEA and SVT
Team Dynamics	1. Did you identify yourself upon arrival or wear an identification badge? (Self-identification)
	a. Yes
	b. No
	c. Not clearly identified but obvious
	2. Did the leader identify her/himself? (Leader Identification)
	a. Yes
	b. No
	c. Not clearly identified but obvious
	3. Did you receive the information needed to perform your role? (Info Received)
	a. Always
	b. Sometimes
	c. Never

Data collection

Data collected included initials, job title, and simulation date, which helped track individual responses to questionnaires at the three different time points. As this simulation training is required annually for all pediatric and PICU nurses, PICU respiratory therapists, and physicians, most of the learners participated twice (i.e., in 2023 and 2024). Participant responses were only tracked from questionnaires 1 to 3 within the same two-hour session.

Outcome

The primary outcome of the study was the improvement in participants’ knowledge, as measured by changes in responses to knowledge-based questions after one and then two simulation sessions. The secondary outcome was the improvement in team behaviors, specifically communication, across the two simulations. Overall, the study sought to measure whether repeated simulation exposure enhanced both clinical knowledge and teamwork performance. 

Data analysis

A total of 99 questionnaires were completed. The participants included 27 pediatric nurses, 24 pediatric intensive care nurses, 4 pediatric intensivists, 18 pediatric hospitalists, 15 respiratory therapists, 8 pharmacists, and 1 non-pediatric staff member. All participants completed the questionnaires, so the results came from a census of all the participants, and sampling was not required.

Data collected over two years were analyzed using McNemar’s and Wilcoxon signed-rank tests. McNemar’s test was used to compare paired binary variables, as with the knowledge questions, for which the responses were correct or incorrect. Effect size was determined based on calculated odds ratios (ORs).

The Wilcoxon signed-rank test was used to compare paired ordinal variables, as with the team dynamic questions, for which the responses were always, sometimes, and never, or yes, not clearly, and no. The effect size was determined by calculating a correlation coefficient using the Z-statistic.

To assess the change in performance, we compared the total number of correct responses across sessions. Results are presented as means, with 95% confidence intervals (CIs). The CIs were calculated using the Student’s t-distribution to account for the sample size (n = 99).

The critical value for each test was 0.05 (α = 0.05). No control variables were analyzed in this study. 

Post-hoc analysis

The Bonferroni correction was applied to account for multiple testing across timepoints. The proportions of correct responses were used to determine question difficulty.

To assess the sensitivity of the study in detecting educational impact, a post-hoc power analysis was performed. For categorical outcomes (e.g., knowledge questions), power was calculated based on observed proportions at each time point. For continuous measures of team dynamics, power was determined using a pre-specified effect size (d = 0.5), corresponding to a 10% improvement target. All power calculations were performed using IBM SPSS Statistics for Windows, Version 31 (Released 2025; IBM Corp., Armonk, NY, USA) with a two-tailed alpha of 0.05.

Internal consistency of the instrument was assessed using Cronbach’s alpha. The alpha for the knowledge questions was determined separately from that of the team dynamic questions, as the question types are inherently different.

To account for participant overlap between the anonymous 2023 and 2024 cohorts, an independent-samples approach, using Fisher’s Exact test for the knowledge questions and the Mann-Whitney U test for the team dynamic questions, was employed. This provided a conservative estimate of baseline comparability.

## Results

A total of 99 questionnaires were completed by 27 pediatric nurses, 24 pediatric intensive care nurses, 4 pediatric intensivists, 18 pediatric hospitalists, 15 respiratory therapists, 8 pharmacists, and 1 non-pediatric staff member.

Application of the Bonferroni adjustment led to an adjusted α = (0.05/15) = 0.003 for knowledge questions and α = (0.05/6) = 0.008 for team behavior questions (Table 2).

**Table 2:** Number and Proportion of Correct Responses to Survey Questions ^a^ Baseline statistically significant increase (p < 0.05). ^b^ Statistically significant increase with Bonferroni adjustment (p < 0.003). ^c^ Statistically significant increase with Bonferroni adjustment (p < 0.008). CPR: cardiopulmonary resuscitation

Questions	Surveys		
	Pre-sim (n = 99)	Post-sim 1 (n = 99)	Post-sim 2 (n = 99)
Knowledge			
Adolescent CPR	63 (64%)	79 (80%)^a^	81 (82%)
Child CPR	78 (79%)	89 (90%)^a^	92 (93%)
Vent Rate With an Advanced Airway	52 (53%)	74 (75%)^a,b^	87 (88%)^a^
CPR Indication	72 (73%)	84 (85%)^a^	89 (90%)
Shockable Rhythms	87 (88%)	89 (90%)	90 (91%)
Team Dynamics			
Self-Identification	-	63 (64%)	86 (87%)^a,c^
Leader Identification	-	72 (73%)	87 (88%)^a,c^
Information Received	-	80 (81%)	93 (94%)^a,c^

Item difficulty is reflected in the proportion of correct responses (Table 2). Questions with proportions of correct responses >80% were considered “very easy,” 70%-79% “easy,” and 50%-69% “moderate.”

Adolescent CPR 

**Figure 2:**
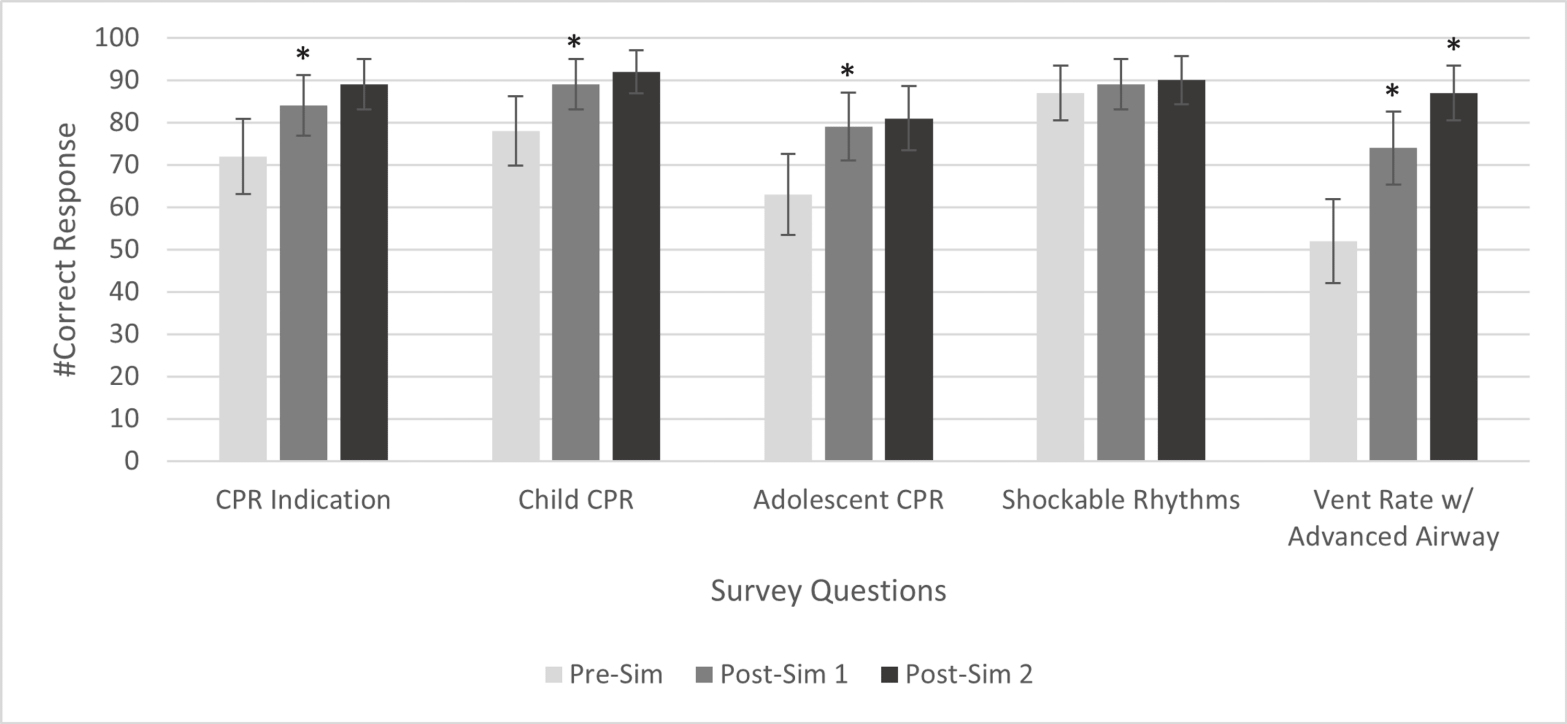
Correct Responses to Knowledge Questions * Statistically significant change compared to the previous survey. Error bars represent 95% confidence intervals. CPR: cardiopulmonary resuscitation; Vent: ventilation; Pre-sim: pre-simulation; Post-sim 1: post-simulation 1; Post-sim 2: post-simulation 2

Correct responses to the question on adolescent CPR are shown in Table 2 and Figure 2. Knowledge of the chest compression-to-ventilation ratio in adolescents with cardiac arrest increased by 16% (n = 16, p = 0.005) after sim 1. The effect size was large (OR = 3.7), indicating that the odds of participant responses changing from incorrect in the pre-sim survey to correct in post-sim 1 were 3.7 times higher than the reverse. There was no statistically significant difference between the proportion of correct answers in post-sims 1 and 2 (p = 0.79).

Child CPR

Correct responses to the question about child CPR are shown in Table 2 and Figure 2. Knowledge of the chest compression-to-ventilation ratio in children improved by 11% (n = 11, p = 0.03). The effect size was large, as indicated by an OR of 3.2. There was no statistical difference between the proportion of correct responses in post-sim 1 and 2 (p = 0.37).

Vent rate with an advanced airway 

Correct responses to the question about ventilation are shown in Table 2 and Figure 2. Knowledge of ventilation rate with an advanced airway increased after both simulations by 22% (n = 22, p < 0.001) and 13% (n = 13, p = 0.004). From pre-sim to post-sim 1, the effect size was large, with an OR of 4.1. From post-sim 1 to post-sim 2, the effect size was also large, with an OR of 5.3.

CPR indication 

Correct responses to the question on CPR indications are shown in Table 2 and Figure 2. Knowledge of indications for CPR in children improved by 12% (n = 12, p = 0.008) from pre-sim to post-sim 1. The effect size was large, with an OR of 5. There was no statistically significant difference between the proportion of correct answers from post-sim 1 to post-sim 2 (p = 0.18).

Shockable rhythms 

Correct responses to the question on shockable rhythms are shown in Table 2 and Figure 2. Statistically significant improvements were not observed in identifying the correct shockable rhythms (p = 0.77 and p = 1.0).

Team dynamics 

**Figure 3:**
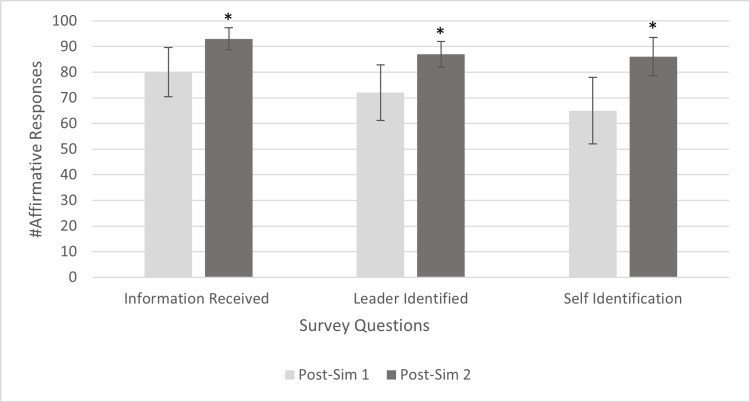
Affirmative Responses to Team Dynamics Questions * Statistically significant change compared to the previous survey. Error bars represent 95% confidence intervals. Post-sim 1: post-simulation 1; Post-sim 2: post-simulation 2

Table 2 and Figure 3 show affirmative responses to questions about team behavior during sim 1 and sim 2. Between post-sims 1 and 2, information was “always” received by 81% (n = 80) of responses and increased to 94% (n = 93) (Z = -3.53, p < 0.001). The correlation coefficient, r = -0.36, represents a small-to-medium effect toward an “always” response.

Self-identification improved from 64% (n = 63) of responses to 87% (n = 86) (Z = -4.05, p < 0.001). The correlation coefficient was r = -0.41, representing a small-to-medium effect toward a “yes” response.

The leader was noted to identify her/himself in 73% (n = 72) of responses in the post-sim 1 survey, and that increased to 88% (n = 87) in the post-sim 2 survey (Z = -3.63, p < 0.001). The correlation coefficient was r = -0.36, indicating a small-to-medium effect toward a “yes” response. The pediatric intensivist responses were excluded from the data analysis for this question, as they were the team leaders.

Internal consistency

Cronbach’s alpha was 0.20 for the pre-sim survey, 0.52 for the post-sim 1 survey, and 0.38 for the post-sim 2 survey. For the team dynamic questions, Cronbach’s alpha was 0.26 for the post-sim 1 survey and 0.30 for the post-sim 2 survey.

Power analysis

The statistical power for categorical items varied depending on the baseline accuracy for each knowledge question and the specific patterns of change observed among participants (Table 3). The majority of observed effects from pre-sim to post-sim 1 surveys demonstrated robust statistical power (>1-β = 0.80).

**Table 3:** Post-hoc Statistical Power for Knowledge Assessment Items Power values were calculated using observed effect sizes for transitions from incorrect to correct responses between surveys. Values >0.80 indicate strong comparisons for detecting a 10% absolute improvement in scores. CPR: cardiopulmonary resuscitation; Pre-sim: pre-simulation; Post-sim 1: post-simulation 1; Post-sim 2: post-simulation 2

Question	Pre-sim/Post-sim 1 Power	Post-sim 1/Post-sim 2 Power
CPR	0.81	0.31
Child CPR	0.83	0.18
Adolescent CPR	0.93	0.08
Shockable Rhythms	0.10	0.06
Vent Rate With an Advanced Airway	0.99	0.89

The power analysis for continuous data (team dynamics) used a fixed 10% improvement target (d = 0.5), which yielded a consistent power of 0.998 across all comparisons.

Baseline (pre-sim) comparison

Pre-sim categorical variables were compared across cohorts using Fisher’s Exact test (Table 4). Pre-sim ordinal responses to team dynamic questions were compared across the cohorts using the Mann-Whitney U test (Table 4).

**Table 4:** Baseline Response Comparison Between 2023 and 2024 Cohorts ^a^ Fisher’s Exact test results from comparing pre-sim performance between the 2023 (n = 44) and 2024 (n = 55) cohorts. ^b^ Analysis of ordinal responses using the Mann-Whitney U test for comparing pre-sim responses between the 2023 and 2024 groups. Pre-sim: pre-simulation; CPR: cardiopulmonary resuscitation; Vent: ventilation

Pre-sim Question	p
CPR	1.0^a^
Child CPR	0.3^a^
Adolescent CPR	0.1^a^
Vent Rate With an Advanced Airway	0.7^a^
Shockable Rhythms	0.5^a^
Self-Identification	0.9 (Z = -0.1)^b^
Leader Identification	0.3 (Z = -1)^b^
Information Received	0.7 (Z = -0.3)^b^

## Discussion

Implementation of iterative practice in multidisciplinary pediatric simulations in a community setting led to knowledge gain, as well as improvement in team behaviors. Cardiac arrest is rare in pediatrics, and even more so in community hospitals, where acuity is lower, but it does occur. Resuscitation of children is complex and requires expertise in age-based CPR guidelines and weight-based medication dosing, both of which necessitate effective leadership and communication. 

Our study showed statistically significant improvement in fpur out of five knowledge-based questions, with large effect sizes reflecting the positive impact of the simulation education. Although this study did not evaluate retention of knowledge over time, the experiential method of gaining (or regaining) this knowledge through simulation and applying it in a subsequent scenario, given a limited amount of education time, is significant. Other studies have also shown improvements in knowledge using simulation. In a meta-analysis and systematic review of 15 studies, high-fidelity simulation increased nursing student knowledge [13]. Similarly, a mock code followed by debriefing increased knowledge of PALS guidelines among pediatric residents [14], who have work-hour restrictions. In this latter study, the addition of didactic sessions did not add further benefit. Multidisciplinary teams have also gained knowledge from simulation-based education [15]. Our study is unique in that improvements are seen among multidisciplinary teams in the inpatient setting of a non-academic community hospital.

While correct responses to the Shockable Rhythms question did not statistically improve after either simulation, the responses to this question started with the highest pretest score (88%), demonstrating participants’ higher knowledge base. Conversely, the participants scored lower on the Vent Rate With an Advanced Airway, Adolescent CPR, CPR Indication, and Child CPR questions (53%, 64%, 73%, and 79%, respectively), leaving more room for improvement. We did not collect data on our participants’ experience level, since years of experience in low-volume and lower-acuity units may not reflect competence in resuscitation. Therefore, similar to novice learners, larger gains were made when the initial score was lower. Studies on trainees most unfamiliar with critical care and neurocritical care (i.e., general surgery residents and maternal-fetal medicine fellows) found the greatest increase in scores after simulation training [9]. By tailoring our simulation education to areas where our participants have the least amount of knowledge, greater gains can be made from the limited education time. 

Another promising finding in this study is that the knowledge gains were made after both simulations in the Vent Rate With an Advanced Airway question. Again, the most room for improvement was for this one question, which had the lowest score on both questionnaires. All the improvements indicate that providing trainees with an opportunity to repeat a scenario is effective in reinforcing gained knowledge, as well as in capturing those learners who need repetition to understand the concept. 

Given that many participants attended a session during both years, a carry-over effect is possible. A sensitivity analysis comparing the baseline knowledge and team dynamic scores of the 2023 and 2024 cohorts showed no statistically significant difference, suggesting that prior exposure did not result in a significant carry-over effect for returning participants. This suggests that participants “reset” to a similar baseline each year. 

When the Bonferroni adjustment was applied to correct for multiple testing, only one knowledge item met this highly stringent threshold. While the four other items did not meet the threshold, the magnitude of effect observed, as evidenced by the ORs, remained significant. From a clinical and pedagogical perspective, the improvements shown with baseline statistical significance remain relevant. In the context of a multidisciplinary simulation, the "cost" of a Type II error - failing to identify a curriculum’s impact on life-saving resuscitation skills - can arguably be as significant as the risk of a Type I error. The analysis focused on targeted, high-priority resuscitation skills established before data collection. Presenting both p-value sets is intended to ensure statistical rigor while preserving the clinical context necessary for future studies.

Leadership and communication are imperative during resuscitation, and their deficiency can lead to medical errors and adverse patient outcomes [16-18]. A study on communication among anesthesiologists in real-life emergencies evaluated methods of call-outs and the prevalence of closed-loop communication, which accounted for 45% of all call-outs [19]. Another hospital-wide study used simulation to examine team behaviors as a baseline in their institution [20]. The proportion of time a shared mental model was established within three minutes was 50%, leadership was verbally established was 6%, and roles/responsibilities were identified was 27% [20]. These studies reflect the gap in team performance and show that robust training is needed to improve team behaviors. Our study collected self-reported behaviors, and while the initial results were not significantly low (lowest 65% self-identified upon arrival), further improvement is needed, and observational data can better characterize team behaviors. Most reported interventions in team behaviors have been through traditional educational methods followed by simulation [18,21,22]. This study relied on the debriefing discussion to review the importance of self-identification, information exchange, and situational awareness, and significant improvements were shown.

Clear leadership is associated with team efficiency and better task and team performance [16]. Leader identification improved in this study, as did information exchange and self-identification, with a small-to-moderate impact of the education session. The improvements may have been due to multiple factors: an increase in, or clearer, self-identification by the leader; clearer communication (e.g., information exchange) among team members; more attention paid by individuals to other team members; and/or increased confidence in sharing/requesting information. Generally, the results reflect more engagement by the team, but future studies - primarily observational - are needed to further examine changes in specific individual behaviors. 

All three team dynamic results remained significant after the Bonferroni adjustment, and the intervention impact was medium to large. Information received demonstrated a highly significant improvement. Self-identification showed the greatest magnitude of change, suggesting that repetitive simulation is particularly effective at reinforcing this behavior. Conversely, leader identification showed the smallest change, suggesting that either the baseline recognition was already high or the specific instructional method requires refinement. Also, there was a medium-sized effect in recognizing the leader. This suggests the simulation effectively taught team members to actively identify the designated leader - a critical skill in resuscitation, where establishing a clear chain of command is essential for effective communication.

The surveys in this pilot study did not demonstrate internal consistency. The knowledge questions were all based on the PALS guidelines and matched the learning objectives. However, the PALS questions did not belong to a single domain, and proficiency in one algorithm did not correlate with proficiency in another. Also, the brevity of the survey, which was designed to be quick, likely limited the alpha value. Another potential reason for the low alpha on the pre-sim survey is the participants’ heterogeneous baseline knowledge. The increase in alpha from the pre-sim survey to the post-sim 1 survey suggests the simulation and debrief led to a shared mental model and aligned the participants’ knowledge. The post-sim 2 survey’s low alpha is likely due to the ceiling effect and minimal variance. As participants’ mastery increased, the reduced variance in scores led to a lower alpha coefficient. The ceiling effect for the knowledge questions is evident in the high baseline scores (>70%) and even higher post-sim 1 scores. Since three of the five questions were already mastered by the majority of participants at the start, and by even more on the post-sim 1 survey, there was limited room for significant improvement following the simulation.

Overall, this study showed that iterative practice can be a useful strategy in enhancing resuscitation knowledge and team performance among practicing providers in a community setting. Like deliberate practice, repetition gives learners the opportunity to show enhanced knowledge and a change in team behavior based on feedback [23]. A previous study among PICU nurses used a similar simulation format with scenario repetition, and, though confidence and knowledge improved when measured at one week and one month post-simulation, the increases in knowledge were not statistically significant [24]. Iterative practice may not aid retention, but it can maximize learning and reviewing in a limited amount of time.

The post-hoc power analysis revealed high statistical power (>0.80) for the majority of core CPR competencies following the initial simulation, and all of the team dynamic performances. Notably, Ventilation Rate With an Advanced Airway demonstrated strong power in both immediate acquisition and retention phases, suggesting high educational durability. Conversely, lower power observed in the retention phase for Adolescent CPR and Child CPR likely reflects a stabilization of scores between post-sim 1 and post-sim 2, rather than a lack of educational impact. The consistently low power for Shockable Rhythm identification suggests this complex cognitive task may require higher-frequency training or a larger sample size to detect significant longitudinal shifts. All the improvements that were >10% and statistically significant (though not by Bonferroni adjustment among most knowledge questions) were adequately powered to detect a meaningful difference.

This study had numerous strengths. The scenarios were held in situ and specifically tailored to the participants’ real-world environment. The structured debriefing sessions were led by a combined team of a pediatric hospitalist and a pediatric intensivist, both certified simulation instructors. By integrating expert-led facilitation, the simulations provided an opportunity for learner self-assessment and focused feedback, which was essential for reinforcing key learning concepts and ensuring effective learning by the participants. The involvement of a multidisciplinary team was another highlight, as their collaboration during a simulated scenario more accurately reflects real-life code events, with the repeated opportunity to improve teamwork and communication. Lastly, inclusion of practicing physicians, which is not common, added to the realism and contributed to the strengthening of the resuscitation teams. 

Several limitations of the study must be acknowledged. First, the data were self-reported by participants and only reflected their knowledge. Observed performance was not evaluated and cannot be determined from the results. Second, leader identification may have been influenced by the individual’s job title (e.g., pediatric intensivist) rather than by explicit self-identifying leadership behaviors. Observed data collection would have better reflected the leader’s performance. Third, there was no control group. Given the limited education time, providing the full experiential curriculum to all participants was prioritized over a randomized design. Fourth, most knowledge question comparisons did not pass the Bonferroni adjustment, making the findings less robust. Furthermore, a power analysis was not done as part of the study design because of the finite number of participants in the department. Fifth, many participants attended twice, warranting concern for a carry-over effect. Additionally, there was low internal consistency. The range of topics (within the PALS guidelines) covered in a small number of items limited the internal consistency. Lastly, the same two debriefers facilitated all the sessions. While that provided consistency in the debriefing quality, slight differences in the material reviewed during debriefs could have impacted the participant responses. A script or checklist to ensure the exact same information was reviewed during the debrief could have been a better reflection of our educational strategy. With a consistent template, variable debriefing techniques through a larger number of facilitators would avoid potentially introducing bias in feedback delivery, and the scale of, and sustainability of, this training model could be improved. 

## Conclusions

As community hospitals face the challenge of maintaining competency for rare pediatric emergencies, iterative simulation practice offers a practical strategy to enhance both individual knowledge and team performance. In this study, multidisciplinary in-situ simulations, with repeated scenarios and structured debriefings, led to significant improvements in key resuscitation knowledge, particularly in areas with lower baseline proficiency, and fostered better communication, leadership, and teamwork.

By providing immediate feedback and the opportunity to apply corrections in a second scenario, iterative practice reinforced skills, clarified roles, and increased engagement among interdisciplinary staff, including those without prior pediatric or PALS training. While this study relied on self-reported knowledge and did not assess long-term retention or observed performance, the findings support iterative simulation as an effective approach to immediate pediatric emergency preparedness. Larger multicenter studies, with objective performance measures, are warranted to further validate and scale this educational strategy. 

## References

[1] Ziv A, Wolpe PR, Small SD, Glick S (2003). Simulation-based medical education: an ethical imperative. Acad Med.

[2] L'Her E, Geeraerts T, Desclefs JP (2020). Simulation-based teaching in critical care, anaesthesia and emergency medicine. Anaesth Crit Care Pain Med.

[3] Binstadt ES, Dahms RA, Carlson AJ, Hegarty CB, Nelson JG (2019). When the learner is the expert: a simulation-based curriculum for emergency medicine faculty. West J Emerg Med.

[4] Whitfill T, Auerbach M, Scherzer DJ, Shi J, Xiang H, Stanley RM (2018). Emergency care for children in the United States: epidemiology and trends over time. J Emerg Med.

[5] Auerbach M, Whitfill T, Gawel M (2016). Differences in the quality of pediatric resuscitative care across a spectrum of emergency departments. JAMA Pediatr.

[6] Abulebda K, Thomas A, Whitfill T, Montgomery EE, Auerbach MA (2021). Simulation training for community emergency preparedness. Pediatr Ann.

[7] Walsh BM, Auerbach MA, Gawel MN, Brown LL, Byrne BJ, Calhoun A (2019). Community-based in situ simulation: bringing simulation to the masses. Adv Simul (Lond).

[8] Elendu C, Amaechi DC, Okatta AU, Amaechi EC, Elendu TC, Ezeh CP, Elendu ID (2024). The impact of simulation-based training in medical education: a review. Medicine (Baltimore).

[9] Boling B, Hardin-Pierce M (2016). The effect of high-fidelity simulation on knowledge and confidence in critical care training: an integrative review. Nurse Educ Pract.

[10] Okuda Y, Bryson EO, DeMaria S Jr, Jacobson L, Quinones J, Shen B, Levine AI (2009). The utility of simulation in medical education: what is the evidence?. Mt Sinai J Med.

[11] McGaghie WC, Issenberg SB, Cohen ER, Barsuk JH, Wayne DB (2011). Does simulation-based medical education with deliberate practice yield better results than traditional clinical education? A meta-analytic comparative review of the evidence. Acad Med.

[12] Eppich W, Cheng A (2015). Promoting excellence and reflective learning in simulation (PEARLS): development and rationale for a blended approach to health care simulation debriefing. Simul Healthc.

[13] Lei YY, Zhu L, Sa YT, Cui XS (2022). Effects of high-fidelity simulation teaching on nursing students' knowledge, professional skills and clinical ability: a meta-analysis and systematic review. Nurse Educ Pract.

[14] Zimmerman E, Wai SS, Hollenbach KA, Cameron MA (2023). Optimizing education during pediatric resident mock code sessions. Pediatr Emerg Care.

[15] McLaughlin C, Barry W, Barin E (2019). Multidisciplinary simulation-based team training for trauma resuscitation: a scoping review. J Surg Educ.

[16] Hunziker S, Johansson AC, Tschan F, Semmer NK, Rock L, Howell MD, Marsch S (2011). Teamwork and leadership in cardiopulmonary resuscitation. J Am Coll Cardiol.

[17] Tourgeman-Bashkin O, Shinar D, Zmora E (2008). Causes of near misses in critical care of neonates and children. Acta Paediatr.

[18] Capella J, Smith S, Philp A (2010). Teamwork training improves the clinical care of trauma patients. J Surg Educ.

[19] Gjøvikli K, Valeberg BT (2023). Closed-loop communication in interprofessional emergency teams: a cross-sectional observation study on the use of closed-loop communication among anesthesia personnel. J Patient Saf.

[20] Ren DM, Abrams A, Banigan M (2022). Evaluation of communication and safety behaviors during hospital-wide code response simulation. Simul Healthc.

[21] Gartland R, Conlon L, Livingston S, Glick JE, Bach G, Abboud ME (2022). Resuscitation leadership training: a simulation curriculum for emergency medicine residents. MedEdPORTAL.

[22] Diaz MC, Dawson K (2020). Impact of simulation-based closed-loop communication training on medical errors in a pediatric emergency department. Am J Med Qual.

[23] Lopreiato JO, Sawyer T (2015). Simulation-based medical education in pediatrics. Acad Pediatr.

[24] Karageorge N, Muckler VC, Toper M, Hueckel R (2020). Using simulation with deliberate practice to improve pediatric ICU nurses’ knowledge, clinical teamwork, and confidence. J Pediatr Nurs.

